# Public Health Genomics: the essential part for good governance in public health

**DOI:** 10.1007/s00038-016-0828-6

**Published:** 2016-05-13

**Authors:** Angela Brand, Nikolaos Evangelatos, Kapaettu Satyamoorthy

**Affiliations:** Faculty of Humanities and Sciences, MERIT (Maastricht Economic and Social Research Institute on Innovation and Technology), Maastricht University, Boschstraat 24, 6211AX Maastricht, The Netherlands; University Clinic for Emergency and Intensive Care Medicine, Paracelsus Medical University (PMU), Nuremberg, Germany; School of Life Sciences, Planetarium Complex, Manipal University, Manipal, India

In view of the importance of the report of the German Academy of Sciences (Leopoldina 2015), discussion on the health impact of Omics was invited (Künzli 2015). We would like to expand the view previously published in this journal (Razum et al. 2015).

Modern accounts of what now constitutes public health have broadened the meaning of the latter, which has come to mean any form of collective action that aims to improve the health of populations (Porter 1998). In that sense, categories, such as medicine, epidemiology and new technologies (to name a few), are species concepts, subjected to a wider genus proximum, public health. In this context, the fascinating field of Omics (genomics, transcriptomics, proteomics, metabolomics, microbiomics, etc.) and Big Data, with its various applications, has already started to revolutionize the landscape of public health, since the beginning of the 21st century (Brand et al. 2008). However, individual scholars and academic institutes have failed to catch sight of the current successes of Omics and Big Data, recognize their entire societal potential and envisage the future (Bayer and Galea 2015; Leopoldina 2015). Moreover, misconceptions of the health impact of Omics have misled scholars and part of the public to doubt their effectiveness and adopt opinions that hinder the implementation of Omics in health systems.

We, on the other hand, argue that Omics are now an organic part of good governance in public health. The idea of modern public health without Omics is simply inconceivable! The reasons for this rely on arguments based on both scientific and societal values.

Going beyond phenotypes, Omics allow for the detection of Mendelian diseases almost to 100 %. And not only that! Identification of resilient individuals opens new highways towards elucidation of genetic disorders and new treatment approaches (Chen et al. 2016). Newborn screening programs are a reality, a current success of Omics, which allows for timely diagnosis even before clinical presentation and enables improved life- and healthspan for affected children. Women with BRCA1 mutations, identified through genetic testing and offered intensive preventive options, are also a reality. Yes, it has not been easy for multifactorial or complex diseases, but with the continued thrust, it will be achievable in the foreseeable future. There are already about 1000 genetic tests for multifactorial disorders and hundreds more are currently being tested, with new approaches, such as the diseasome, facilitating the conceptualization of the genome-disease interactions. For the patients with potentially life threatening multifactorial disorders, such as cardiac channelopathies and cardiomyopathies, timely diagnosis and treatment options, are already becoming a reality.

Apart from diagnosis, Omics have also been changing the way we approach treatment. An ever growing list of genetic polymorphisms is now used as a basis for predicting response to more than 100 drugs. Pharmacogenomics allow for the identification of patients most likely to respond to a certain treatment, enable tailoring of drug dosage and minimize adverse drug reactions. The importance of pharmacogenomics becomes even more evident if we take into account that 38–75 % of patients are estimated to be unresponsive to drugs (Lehrach 2015).

A third pillar of Omics applications is related to their ability to fully exploit the potential of traditional public health interventions. Although a relatively new approach, genetic profiling enables population stratification as to their genetic predisposition and guides prevention programs accordingly, thus rendering them more effective in terms of cost and benefit. In an uncertain global economic landscape, cost effectiveness of any public health intervention is of major importance and Omics can help us both optimize the outcome and reduce the costs of our interventions.

Effective screening and prevention programs, early diagnosis, proper treatment and minimization of costs enabled by Omics are obvious benefits for both patients and health care systems. Is that not, after all, the essence of successful public health programs?

The contribution of Omics to public health transcends conventional academic and industrial barriers and transforms public health in ways that traditional approaches simply cannot. Apart from public health interventions, we also need effective drugs in our armamentarium. In an era when the rate of new drug approvals stagnates, drug development has moved from the classical clinical development approach of the many, sequential, distinct phases, to a more integrative approach with adaptive clinical trial designs. This is a pure effect of the implementation of Omics approaches and has already started delivering its first results (Schulthess et al. 2015).

Furthermore, and perhaps even more excitingly, knowledge in the form of integrated and socially meaningful Omic data has increasingly been conceptualized as public good. Sharing of Omic data from clinical trials enhances public confidence in clinical trials results and fosters the innovation capability of the biopharmaceutical industry, thus leading to the production of targeted drugs that allow for the realization of personalized medicine. However, the social and economic externalities of Omics do not stop here. Health Data Cooperatives enabled by Omics, such as MIDATA.coop, empower citizens to the benefit of individuals, healthcare systems and the society (Hafen et al. 2014).

From the above, it is obvious that we are in the middle of a major paradigm shift that leads us towards a ‘systems thinking’ as to disease etiology, prevention and treatment (Brand 2011). However, we are confronted with challenges that need to be addressed. It is clear that we will have to go through a period of ‘normal science’ (in the Kuhnian sense) (Kuhn 1962) which will shed light on the constant interactions between the genome and the phenome (Houle et al. 2010). Moreover, the impact of Omics on public health cannot be fully realized, unless their implementation and integration in health care systems are fully deployed. A critical mass has to be reached…

Effective implementation of Omics-based technologies requires coordinated actions and appropriate modifications of public health and health governance systems at all levels (Brand et al. 2012). Starting in Germany and followed by other European Member States, significant work towards this direction has been done by the Public Health Genomics European Network (PHGEN) (http://www.phgen.eu) and guidelines have been developed to ensure the maximum impact on health and economic growth in the health sector.

It is incontrovertible that the traditional approaches based on epidemiology, such as tobacco control, have conferred significant advantages in the health of populations. However, they seem to have reached their capacity as to the added value they can confer, at least to parts of the population which have already benefited from such interventions (e.g., people who have quitted smoking). On the other hand, Omics have been providing sound evidence of their potential, as an essential part of good governance, to radically transform the landscape in public health. Unless the skepticism of traditional public health programs towards Omics can present us a sustainable alternative to Omics, there is no other way, beneficial to the population, for them, but to welcome new partners and new approaches to common scopes. The high relevance of the field of Omics for public health and health policy has recently also been expressed by European policymakers. In the Council conclusions on personalized medicine for patients, which has been developed under the Luxembourg Presidency in 2015 (Council 2015), the step-by-step implementation of Public Health Genomics, both at European Union and national level, is encouraged. Public Health Genomics offers a debate platform, where a productive ‘innovation diplomacy’ can find place—we welcome constructive contributions!
